# Group-delivered cognitive behavioural therapy versus waiting list in the treatment of insomnia in primary care: study protocol for a pragmatic, multicentre randomized controlled trial

**DOI:** 10.1186/s12875-023-02018-4

**Published:** 2023-03-02

**Authors:** Maria Hrozanova, Ingebrigt Meisingset, Håvard Kallestad, Ståle Pallesen, Anne Lovise Nordstoga, Eivind Schjelderup Skarpsno

**Affiliations:** 1 Department of Public Health and Nursing, Norwegian University of Science and Technology, Trondheim, Norway; 2 Unit for Physiotherapy Services, Trondheim Municipality, Trondheim, Norway; 3 Department of Mental Health, Norwegian University of Science and Technology, Trondheim, Norway; 4 Department of Mental Health Care, St. Olavs Hospital, Trondheim, Norway; 5 Department of Psychosocial Science, University of Bergen, Bergen, Norway; 6 Norwegian Competence Center for Sleep Disorders, Haukeland University Hospital, Bergen, Norway; 7 Department of Neuromedicine and Movement Science, Norwegian University of Science and Technology, Trondheim, Norway; 8 Department of Physical Medicine and Rehabilitation, St. Olavs Hospital, Trondheim, Norway; 9 Department of Neurology and Clinical Neurophysiology, St. Olavs Hospital, Trondheim, Norway

**Keywords:** Sleep problems, Insomnia disorder, Sleeplessness, Primary healthcare, Adults, Psychological treatment

## Abstract

**Background:**

Insomnia is common in the general population and is a risk factor for ill-health, which highlights the importance of treating insomnia effectively and cost-efficiently. Cognitive-behavioural therapy for insomnia (CBT-I) is recommended as first-line treatment due to its long-term effectiveness and few side-effects, but its availability is limited. The aim of this pragmatic, multicentre randomized controlled trial is to investigate the effectiveness of group-delivered CBT-I in primary care compared to a waiting-list control group.

**Methods:**

A pragmatic multicentre randomized controlled trial will be conducted with about 300 participants recruited across 26 Healthy Life Centres in Norway. Participants will complete online screening and provide consent before enrolment. Those who meet the eligibility criteria will be randomized to a group-delivered CBT-I or to a waiting list according to a 2:1 ratio. The intervention consists of four two-hour sessions. Assessments will be performed at baseline, 4 weeks, 3- and 6 months post-intervention, respectively. The primary outcome is self-reported insomnia severity at 3 months post-intervention. Secondary outcomes include health-related quality of life, fatigue, mental distress, dysfunctional beliefs and attitudes about sleep, sleep reactivity, 7-day sleep diaries, and data obtained from national health registries (sick leave, use of relevant prescribed medications, healthcare utilization). Exploratory analyses will identify factors influencing treatment effectiveness, and we will conduct a mixed-method process evaluation to identify facilitators and barriers of participants’ treatment adherence. The study protocol was approved by the Regional Committee for Medical and Health Research ethics in Mid-Norway (ID 465241).

**Discussion:**

This large-scale pragmatic trial will investigate the effectiveness of group-delivered cognitive behavioural therapy versus waiting list in the treatment of insomnia, generating findings that are generalizable to day-to-day treatment of insomnia in interdisciplinary primary care services. The trial will identify those who would benefit from the group-delivered therapy, and will investigate the rates of sick leave, medication use, and healthcare utilization among adults who undergo the group-delivered therapy.

**Trial registration:**

The trial was retrospectively registered in the ISRCTN registry (ISRCTN16185698).

**Supplementary Information:**

The online version contains supplementary material available at 10.1186/s12875-023-02018-4.

## Background

Insomnia is the most common sleep disorder in the general population and in clinical practice [1], with ~ 10% of adults reporting chronic insomnia in agreement with the current diagnostic classification system [2] and seems to be on the rise [3]. Among patients seeking primary care, as many as 50–70% report disturbed sleep [4, 5]. Cognitive behavioural therapy for insomnia (CBT-I) is considered most effective for long-term alleviation of chronic insomnia [6–8], but administration of CBT-I is limited by a lack of trained therapists and long waiting lists [9] (i.e., up to 1 year in Norway). To increase the availability of CBT-I, a group-delivered treatment was developed by the Norwegian Health Directorate. The treatment is based on core CBT-I principles including sleep hygiene, stimulus control, sleep restriction, cognitive restructuring, and relaxation training. Group-delivered CBT-I may complement individual CBT-I and relieve pressure on primary care, as group size may include up to 15 individuals at the same time. Although group-delivered CBT-I has already been implemented in several municipalities in Norway, its effectiveness has never been evaluated. Therefore, the objective of this study is to investigate the effectiveness of group-delivered CBT-I in Norwegian primary care.

It is of interest to identify factors that may influence the therapeutic response of group-delivered CBT-I. Up to 40% of insomnia patients do not adequately respond to CBT-I [10], likely due to insomnia heterogeneity [10–12]. For instance, insomnia patients with evening chronotype, unhelpful beliefs about sleep, short sleep, comorbidity, or high reactivity to stress may have a blunted response to CBT-I [12–15], possibly due to the influence of overactive neurobiological and psychological systems in insomnia pathophysiology [16]. Adherence to the therapeutic regime may also predict outcome [17]. Thus, a secondary objective of this study is to conduct exploratory analyses to identify factors influencing the effectiveness of group-delivered CBT-I.

### Aims

The primary aim is to conduct a pragmatic, multicentre randomized controlled trial (RCT) to investigate the effectiveness of group-delivered CBT-I in primary care on insomnia severity at 3 months post-treatment. Secondary aims include:

i. Investigate the effectiveness of group-delivered CBT-I on health-related quality of life, fatigue, mental distress, and sleep diary data at 3- and 6-months post-treatment.
ii. Investigate whether potential treatment moderators (e.g., chronotype, reactivity to stress, duration of insomnia, length of prior insomnia treatment, treatment group size, physical activity) influence the effectiveness of group-delivered CBT-I.
iii. Compare sick leave days, use of relevant prescribed medication (e.g., psychotropics, sedatives) and healthcare resource utilization, utilizing national registry data at pre-treatment vs. 1- and 2-years post-treatment.
iv. Conduct exploratory mediation analyses to identify mechanisms behind change in the primary and secondary outcomes, focusing on psychological measures of beliefs about sleep, reactivity to stress, and sleep-related self-efficacy as potential mediators.
v. Conduct a mixed-method process evaluation to assess facilitators and barriers of participants’ treatment adherence.

## Methods and analysis

This RCT protocol follows the Standard Protocol Items for Randomized Trials (SPIRIT) statement guidelines [18] and all methods were carried out with relevant guidelines and regulations. The trial is registered in the ISRCTN registry (ISRCTN16185698). Supplementary files include the SPIRIT chart (S1), SPIRIT checklist (S2), and WHO Trial Registration Data Set (S3).

### Study design and setting

**Fig. 1:**
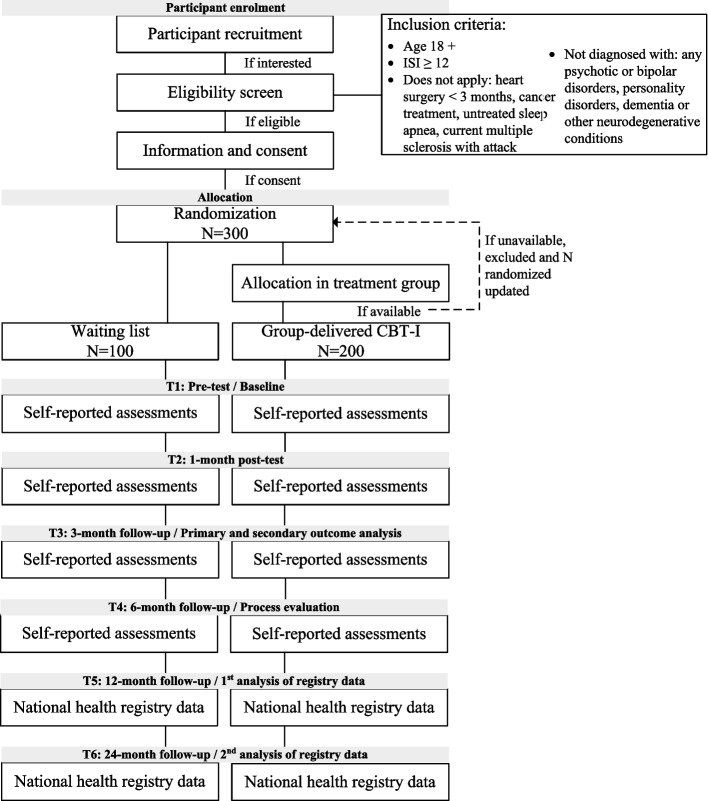
Planned study design of the pragmatic, multicentre randomized controlled trial investigating the effectiveness of group-delivered CBT-I on insomnia severity in primary care. ISI = Insomnia Severity Index, CBT-I = cognitive behavioural therapy for insomnia

This is a pragmatic, multicentre RCT that will investigate whether group-delivered CBT-I, compared with a waiting list, reduces insomnia severity. The group-delivered CBT-I will be offered at the public, interdisciplinary primary care Healthy Life Centres in Norway. The group-delivered CBT-I investigated in this study is an existing treatment option at the Healthy Life Centres for adults with insomnia symptoms. A flow chart of the planned study design of the RCT is shown in Fig. 1.

### Participants

#### Recruitment

Recruitment of centres lasted from April 2022 to September 2022. Of the 280 Healthy Life Centres in Norway, 49 offer the group-delivered CBT-I treatment. All 49 centres were contacted via e-mail with information about the research project. Of the 49 centres, 26 have agreed to participate in the research project (53% response rate).

Recruitment of participants with insomnia commenced in August 2022. Participants are recruited using advertisements in newspapers and news websites, social media, webpages for the involved municipalities and Norwegian University of Science and Technology (NTNU), through the Healthy Life Centres, and poster advertisements in waiting rooms of general practitioners and physiotherapists. Healthcare personnel will be informed about the RCT using relevant communication channels and may refer patients to the group-delivered CBT-I treatment.

#### Eligibility and procedure

All adults interested in participation will enter a secure online screening portal, through a link on the project’s webpage. The screening portal includes a series of self-rating questions pertaining to the eligibility criteria. Inclusion criteria include age ≥ 18 and Insomnia Severity Index (ISI) score ≥ 12, indicating insomnia symptoms with a significant impact on the individual [19]. Since this is a pragmatic trial, we will only exclude adults with diagnosed major psychiatric, somatic, or neurological disorders for which CBT-I may be contraindicated (see Fig. 1). All potential participants will have to confirm their proficiency in the Norwegian language.

The researchers will evaluate inclusion criteria of each potential participant. Eligible participants will be sent a digital consent form. After consenting, participants will be randomized, and thereafter informed of allocation outcome. Participants in the treatment group will be contacted by the respective Healthy Life Centre employees about the next available treatment group. If they cannot participate (i.e., the time or location of treatment group does not align with participants’ schedule), they will be excluded. All participants will obtain invitations to complete questionnaire batteries and sleep diaries via e-mail at four measurement timepoints (see Fig. 1). Non-responders will receive reminders via SMS or phone call. Those who still do not respond will be followed up by phone and asked instead to report only on the primary outcome measure (ISI).

#### Randomization

Participants will be randomized to the intervention and control group according to a 2:1 ratio. Thus, groups will be formed quicker with more intervention participants, allowing more CBT-I groups to be formed during the limited timeframe of the trial. Unequal randomization may also facilitate recruitment, as participants may be more likely to enrol if they perceive their chance of receiving the intervention to be greater than being placed on the waiting list. Many participants seek out the treatment themselves and reducing the number of waiting list participants is also desirable for ethical purposes. Finally, more intervention group participants will ensure the conduct of planned moderation and exploratory mediation analyses with increased power. Unequal randomization in similar research has previously been recommended [20–22].

Randomization will be performed by the project leader in a secure digital platform for multicentre clinical studies, eForsk. eForsk is a standalone data management system used for electronic self-report data, developed by the Central Norway Regional Health Authority IT department. The randomization will be based on a computerized allocation sequence stratified on each of the participating centres. This will be generated by a third party, the Clinical Research Unit at the Faculty of Medicine and Health Sciences at NTNU. The project leader will be blinded for the block sizes and the allocation sequence. The project leader and research team will thus not influence the randomization process in any way. An overview of the allocation sequence can be provided by the Clinical Research Unit upon request. Participants and the Healthy Life Centre employees will not be masked to the participants’ group assignment.

### Interventions

#### Group-delivered CBT-I

Participants in the intervention arm will be offered a group-delivered CBT-I treatment developed by the Norwegian Health Directorate in collaboration with the Norwegian Competence Centre for Sleep Disorders. The intervention will be provided repeatedly at the 26 centres recruited for this study. Group size varies between centres, from 5 to 15 participants per treatment group. The treatment is a first line, low-threshold service for sleep problems in primary care without the need of referral.

The intervention will follow the standardized CBT-I program, with some adaptations based on input from researchers, clinicians, and experiences of the user group. Components of the intervention are presented in Table 1.

**Table 1:** Components of the investigated group-delivered cognitive behavioural therapy for insomnia

Session	Educational components	Group components	Homework
1	Sleep, its functions and sleep/wake regulation Prevalence, symptoms, and consequences of insomnia Using the sleep diary Relaxation technique: Herbert Benson’s relaxation response	Participants meet each other and talk about their experiences with insomnia Participants’ course expectations	Identify three sleep-related habits to target during treatment Fill out sleep diary Fill out Sleep Hygiene Index
2	Sleep hygiene advice Stimulus control Relaxation technique: Progressive muscle relaxation	Participants’ experiences with sleep diary Learning outcomes so far Implemented changes so far	Fill out sleep diary Fill out the Negative Beliefs and Attitudes about Sleep Apply sleep hygiene advice Calculate sleep efficiency in preparation for sleep restriction
3	Dysfunctional beliefs about sleep: Identification and change Principles of sleep restriction	Implemented changes and their effect so far	Fill out sleep diary Fill out an overview of how to challenge own negative thoughts about sleep Apply sleep restriction
4	Management and maintenance of own sleep Motivation for lasting change	Implemented changes and their effect so far Alternatives to dysfunctional beliefs about sleep Adapting the learning outcomes to changing life-circumstances	

The intervention will be conducted by Healthy Life Centres employees who are physiotherapists with extensive experience and knowledge of the treatment. They have participated in a course about delivering the intervention, organized by the Norwegian Health Directorate. All have also participated in an in-person information day, held prior to the start of the RCT, where the research project and the relevant adaptations to the intervention were presented in detail, to ensure a common knowledge basis for all instructors. The meeting was recorded, and distributed to the Healthy Life Centres employees for reference.

#### Control group

Participants in the control group will be placed on a waiting list for 6 months. They will not be restricted from seeking other forms of treatment during the waiting period, nor from using sleep medications. After the waiting period, they will have the possibility to participate in the treatment at their Healthy Life Centre, which will be offered to them by a Healthy Life Centre employee during the waiting period.

### Assessments

Assessments will be carried out at 6 different time points during the RCT: T1, prior to the intervention; T2, immediately post-intervention; T3, 3 months post-intervention (primary outcome measurement); T4, 6 months post-intervention; T5, 1-year post-intervention; and T6, 2 years post-intervention. The primary outcome is self-reported insomnia severity assessed by the ISI. Several other outcomes will be used in analyses of secondary outcomes, moderators and exploratory mediators, resource use and work productivity, and in a mixed-method process evaluation, described below. Descriptions of all measures, objectives of their use, collected, the participant group receiving them, and the objectives of their use, are provided in Table 2.

**Table 2:** Overview of the assessments utilized in the randomized controlled trial

Objective	Measure	Measurement timepoint	Participant group
Demographic information	Self-rated custom questions	T1	All
Primary outcome	Insomnia severity (Insomnia severity index)	Screening, T1, T2, T3, T4	
Secondary outcomes	Health-related quality of life (EuroQol EQ5D-5L)	T1, T2, T3, T4	
	Fatigue (Chalder Fatigue Scale)	T1, T2, T3, T4	
	Mental distress (Hopkins Symptoms Checklist)	T1, T2, T3, T4	
Moderator	Chronotype (Brief Horne-Östberg Morningness-Eveningness Questionnaire)	T1, T2, T3, T4	
Mediators	Unhelpful beliefs about sleep (Dysfunctional Beliefs and Attitudes about Sleep questionnaire-16)	T1, T2, T3, T4	
	Sleep reactivity (Ford Insomnia Response to Stress Test)	T1, T2, T3, T4	
Subjective assessment of sleep patterns	7-day sleep diary	T1, T2, T3, T4	
Mixed-method process evaluation of facilitators and barriers of participants’ treatment adherence in group-delivered CBT-I	Motivation for treatment (Nijmegen Motivation List 2)	T1	~ 60 intervention group participants
	Treatment credibility (Credibility / Expectancy Questionnaire)	T1	
	Adherence to CBT-I components (Treatment Components Adherence Scale)	T3	
	Evaluation of treatment (CBT-I evaluation scale)	T3	
	Individual semi-structured interviews	T2, T3	10–12 intervention group participants
Evaluation of sick leave rates, medication use, and healthcare services utilization	Norwegian Patient Registry	T5, T6	All intervention group participants
	Norwegian Prescription Database	T5, T6	
	National Insurance Administration	T5, T6	

#### Demographic questionnaire

The demographic questionnaire includes questions on age, sex, pregnancy in women, highest achieved education level, marital status, children in the household below and over 6 years, occupational status and work ability, shift work, weight and height, physical activity, frequency of muscle pain, use of alcohol and illegal substances, as well as questions about recruitment location, duration of participants’ sleep problems and prior insomnia treatment.

#### Sleep measures

##### *Primary outcome measure*

Insomnia Severity Index (ISI): ISI is a recommended outcome measure in sleep research [23], considered as the gold standard self-report measure of insomnia severity in clinical practice. Its seven items assess symptoms of insomnia such as difficulty falling or staying asleep, satisfaction with sleep, and degree of impairment with daytime functioning. The composite score ranges from 0 to 28 and represents no clinical insomnia (0 to 7), sub-threshold insomnia (8 to 14), insomnia of moderate severity (15 to 21), and severe insomnia (22 to 28). ISI has been validated extensively and has proven sensitive to therapeutic changes [23].

##### *Other sleep measures*

Digital sleep diary: Participants will be asked to provide daily subjective estimates of their sleep for 7 consecutive days in a validated sleep diary [24], documenting their daily function, time of entering bed and lights off, the approximate sleep onset latency after lights off, frequency of and length of awakenings, final wake up and rise time, and subjective sleep quality.

#### Physical and mental health measures

EuroQol EQ5D-5L: The EuroQol EQ5D-5L questionnaire [25] will be used to assess health-related quality of life. Its five items assess mobility, self-care, usual activities, pain/discomfort, and anxiety/depression. Items are scored on a 5-point scale, following the format ‘no problems’, ‘slight problems’, ‘moderate problems’, ‘severe problems’, and ‘unable to’/’extreme problems’ for all items.

Chalder Fatigue Scale: The Chalder Fatigue Scale [26] measures the extent and severity of fatigue in fatiguing illnesses. We will use the Norwegian 13-item version. The items are answered on a 4-point scale ranging from asymptomatic to maximum symptomology, such as ‘Better than usual’, ‘No worse than usual’, ‘Worse than usual’ and ‘Much worse than usual’. The global score may be divided into two dimensions: physical fatigue (items 1–7) and psychological fatigue (items 8–11).

Hopkins Symptom Check List: Mental distress will be measured using the Hopkins Symptom Check List-5 [27], which consists of 5 items assessing symptoms of depression and anxiety. The items are answered on a 4-point scale, and range from ‘Not at all’ to ‘Extremely’.

#### Process evaluation

Nijmegen Motivation List 2: The Nijmegen Motivation List 2 [28] will be used to investigate different aspects of participants’ motivation for treatment: Preparedness and active participation, distress as a consequence of participants’ problems, and doubt and reserved attitude towards treatment, its requirements, or the possibilities of benefiting from it. The questionnaire consists of 34 items, scored on a 6-point Likert scale from 1, ‘Not at all applicable’ to 6, ‘Very applicable’.

Credibility / Expectancy Questionnaire: The Credibility / Expectancy Questionnaire [29] asks about the improvements that participants believe will be achieved as a result of treatment, and how believable, convincing, and logical the treatment seems. Its 6 items are scored on a numerical rating scale from 1, ‘Not at all logical’ to 9, ‘Very logical’.

Treatment Components Adherence Scale: The adherence to key behavioural and cognitive CBT-I treatment components, and their perceived benefits, will be assessed with the Treatment Components Adherence Scale [30]. Its 23 items are scored on 4-point Likert scale from 1, ‘Followed rarely or not at all’ to 4, ‘Followed consistently’.

Treatment evaluation scale: The treatment evaluation sheet [31] asks about the relevance, implementation and perceived safety of treatment. The sheet consists of 11 items scored on a 5-point Likert scale from 1, “Not satisfactory” to 5, “Very good”.

Suggestions for improvements: Participants in the intervention group will be asked, in writing, to answer open-ended questions about what worked particularly well and poorly for them during the CBT-I treatment, and their suggestions for treatment improvements.

Qualitative study: A qualitative study will explore facilitators and barriers of participants’ treatment adherence. Individual semi-structured interviews will be conducted at T2 and T3 with 10–12 participants. The interviews will be based on an interview guide developed to answer: “What motivated you to commence and persist with group-delivered CBT-I in primary care?” (adapted from [32]). Usefulness of treatment aspects and the instigated, upheld and/or abandoned changes will be investigated, as well as the processes or experiences that have influenced participants’ actions in behaviour change, or lack thereof. If possible, participants who dropped out from treatment will be interviewed to investigate the reasons for dropping out and what could have helped them finish treatment. The interview guide consists of nine open-ended thematic questions and allows for new questions to be formulated and added to the interview schedule throughout data collection. Question order will be kept flexible, following the participants’ thoughts and narratives. The interviewer will probe areas of interest to the problem statement through all stages. The interviews will be analysed according to the Framework Method, a method used in multi-disciplinary health research teams [33].

#### Potential moderators and mediators

Brief Horne-Östberg Morningness-Eveningness Questionnaire: The reduced Horne-Östberg-Morningness-Eveningness Questionnaire is a widely used measure of the morningness-eveningness dimension [34]. Its five items refer to rising time, peak time, retiring time, morning freshness, and self-evaluated chronotype. A composite score from 4 to 25. Higher levels of morningness are reflected by higher scores.

Dysfunctional Beliefs and Attitudes about Sleep: Participants’ sleep-related cognitions will be measured using the questionnaire Dysfunctional Beliefs and Attitudes about Sleep-16 [35], which consists of 16 items assessing sleep-related cognitions (e.g., faulty beliefs an appraisals, unrealistic expectations, perceptual and attentional bias).

Ford Insomnia Response to Stress Scale: Sleep reactivity will be measured using the Ford Insomnia Response to Stress Scale [36]. Its 9 items assess the vulnerability to situational insomnia under 9 different stressful conditions (i.e., sleep reactivity). The items are scored on a 4-point scale, ranging from ‘Not likely’ to ‘Very likely’.

#### Resource use and work productivity

We will access national health registry data at T5 and T6, to collect information on the short- and medium-term impact of group-delivered CBT-I on rates of sick leave (accessed from the National Insurance Administration), as well as relevant medication (obtained from the Norwegian Prescription Database) and health resource utilization (obtained from the Norwegian Patient Registry). Linkage to the registry data will be based on the social security number of each participant.

#### Sample size

The average observed change in ISI in published RCTs on the effectiveness of group-delivered CBT-I [37–41] from baseline to post-test was 6.2 points in the intervention group, compared to a 1.4-point change in the control group. This indicates a large Cohen’s *d* effect size of 0.86. However, we chose the medium Cohen’s *d* effect size of 0.50 for the sample size calculation, mostly due to the differences in the number of given CBT-I sessions across studies. Treatment in this RCT includes 4 sessions, while other reviewed studies include 5 [37, 38, 41] and 8 [39, 40] sessions, respectively. It is reasonable to assume that treatment effectiveness may decrease with fewer number of sessions. Further, since this is a pragmatic trial, we will not prevent the control group participants from seeking other forms of treatment during the RCT, nor it is a requirement that the participants do not use sleep medications. Thus, treatment effectiveness in this trial may be assumed to be lower than in the aforementioned RCTs.

A power analysis was carried out using a two-tailed t-test with 5% alpha level and 90% power to detect a medium Cohen’s *d* effect size of 0.50, with an allocation ratio of 2:1 (G*Power, version 3.1.9.6) [42]. The needed sample size was 192 in total. Additionally, to have sufficient power for moderation analyses of chronotype, we carried out a two-tailed t-test with 5% alpha level and 80% power to detect a small to medium Cohen’s *d* effect size of 0.40, with an allocation ratio of 2:1, and accounting for a 30% attrition rate based on dropout rates reported by previous studies [43–46]. The final needed sample size was 292 participants (98 in the control group, 194 in intervention group). Since missing data are unlikely to be an issue when using national registers, we did not undertake any statistical power calculations related to these analyses.

### Data management and auditing

All self-reported health information will be distributed and collected using the previously described eForsk platform. Upon randomization of each participant, researchers will share an interactive document including participant name, date of birth, phone number and randomization outcome, with the Healthy Life Centre employee to which the participant belongs. In the document, the Healthy Life Centre employee will fill out the date for treatment initiation. Researchers will use this date to plan the distribution of self-report questionnaires. Each Healthy Life Centre will have their own interactive document. Each document will be password protected and shared only with the Healthy Life Centre employees involved in the study.

Upon completion of data collection, an anonymized database will be exported from eForsk, sufficiently encrypted, and stored within the NTNU file system. The participant identification key will be confidential and stored within the eForsk database, with only the project leader having access. All data storage and analysis will be conducted according to the regulations by NTNU and will follow the General Data Protection Regulation. Data will not be disclosed to researchers outside the project or transferred to other countries. A data monitoring committee will not be involved in this study, due to the minimal risks associated with the investigated intervention.

Trial conduct will be audited in line with NTNU’s guidelines. This entails the possibility of an internal quality-assuring control where project information, data management systems, formal approvals, protocol amendments, etc. may be audited. Moreover, two independent project members are employed to follow data collection and management, and to provide support to any participants experiencing issues with the data collection. These project members have regular contact with the Healthy Life Centres participating in the trial. Both the project members and the project leader are informed about withdrawals from the study, and possible adverse events are reported to the Healthy Life Centre employees by the participants.

#### Statistical analysis plan

A detailed statistical analysis plan will be published before unblinding of study data. Descriptive statistics will be presented stratified by group allocation. Categorical and binary variables will be summarized as counts and percentages, while continuous variables as means and standard deviations or medians and interquartile range, as appropriate.

The primary outcome will be analyzed using the intention-to-treat principle. Linear mixed models will be used to estimate the mean differences between T1 and T3 in ISI with 95% confidence intervals between the two groups. For continuous secondary outcomes, linear mixed models will be utilized, while binary secondary outcomes (e.g., clinically relevant change in ISI) will be analyzed using logistic mixed models. Models will include time, group, time–group interaction and baseline covariates. Since we expect that some intervention group participants will miss some treatment sessions, per-protocol analyses will be used for individuals who complete ≥ 3 sessions. Missingness patterns will be investigated, and pattern-mixture models will be used to simulate situations where the data are not missing at random. Interim analyses will not be carried out, due to limited expected adverse effects of the treatment, and because all intervention group participants are followed up by the Healthy Life Centre employees during the treatment.

Moderation analyses will be conducted to investigate whether demographics (e.g., age, sex) chronotype, treatment group size, physical activity, length of prior insomnia treatment, and duration of insomnia moderate the effectiveness of group-delivered CBT-I on primary and secondary outcomes. Exploratory mediation analyses will be conducted to investigate mechanisms of change in the primary and secondary outcomes, focusing on psychological measures of beliefs about sleep, reactivity to stress, and sleep-related self-efficacy.

### Monitoring

The time and effort necessary for the self-reported assessments may impose some participant burden. To increase compliance and alleviate participant burden, control group participants will be offered a monetary compensation of 150 NOK (10 NOK ~ 1 EUR) if they report on all questionnaires and ≥ 4 sleep diaries at timepoints T1, T2, T3 and T4, respectively. Moreover, participation in the group-delivered CBT-I is typically subject to a fee (varying from 200–550 NOK between the Healthy Life Centres), but intervention group participants will be exempted from paying this fee.

The study will impose participant focus on insomnia, own sleep, and health-related consequences. For some, this may be perceived as stressful or worrying. However, all participants are seeking out insomnia treatment, and most likely already have an increased focus on these aspects in their daily lives. The harm associated with participation is therefore deemed acceptable. Participants who contact the research team or the Healthy Life centre employees about participation burden will be followed-up individually. All participants will be informed about the possibility to terminate participation in the study for any reason.

Control group participants are placed on a 6-month waiting list before they can participate in the treatment. This waiting time is deemed acceptable, as the usual waiting time for the treatment varies from 1 to 6 months, depending on the centre (i.e., centres in more sparsely populated municipalities have longer waiting periods, as they organize fewer treatment groups). Likewise, some intervention group participants may experience a waiting time of up to 6 months before they can begin treatment. This reflects natural capacity limitations. To alleviate the waiting time, multiple centres will be organizing more frequent treatment groups, and waiting times over 3 months for the intervention group participants will rarely occur. Relevant for all study participants is the notion that participation in the RCT will not be a prerequisite for attending the group-delivered CBT-I treatment at the Healthy Life Centers – those who decline to take part in the RCT will still be able to attend the treatment.

### Patient and public involvement

Multiple representatives of the Healthy Life Centers, and a representative of a mental health user group, were involved during study conceptualization, design, and conduct. During study design, 5 earlier treatment participants were asked to test and evaluate the self-reported assessments. To user groups, and public and patient stake holders, findings will be disseminated using user-friendly outputs.

## Discussion

This nation-wide, pragmatic RCT with 26 participating centres will investigate the effectiveness of group-delivered cognitive behavioural therapy versus waiting list in the treatment of insomnia. Primary outcome is insomnia severity at 3 months post-treatment. While this trial will generate novel findings about insomnia treatment in interdisciplinary primary care services, there are also a number of limitations. First, as the recruitment strategy is based on voluntary participation and self-referral, there is a possibility of participant self-selection bias. Second, even though all Healthy Life centres employees participating in the trial had prior experience with delivering the intervention, and all attended an information meeting about the research project to ensure a common knowledge basis for all instructors, there is a lack of treatment integrity investigation at the 26 participating centres. Thus, it is possible that there may be quality differences in treatment administration between the participating centres. Third, no objective measurements of sleep are employed in the trial. Fourth, since data at the first four measurement timepoints is based on self-report, there is a possibility of missing data and sample attrition. Participants who do not adhere to the data collection will be followed up to mitigate some of the associated risk. Lastly, the 6-month waiting period for the control group may lead to dropout from the trial or use of other treatment options during the intervention.

## Supplementary Information

**Additional file 1.**

**Additional file 2.**

**Additional file 3.**

## Data Availability

Not applicable.

## Abbreviations

CBT-I: Cognitive behavioural therapy for insomnia
RCT: Randomized controlled trial
SPIRIT: Standard Protocol Items for Randomized Trials
NTNU: Norwegian University of Science and Technology
ISI: Insomnia Severity Index
