# Supplementary material for: Group-delivered cognitive behavioural therapy versus waiting list in the treatment of insomnia in primary care: study protocol for a pragmatic, multicentre randomized controlled trial
Source: BMC Prim Care. 2023 Mar 2;24:61. doi: 10.1186/s12875-023-02018-4 (PMC9979487; doi:10.1186/s12875-023-02018-4)
Supplement: Supplementary file 1 — Additional file 1. [file 12875_2023_2018_MOESM1_ESM.docx]

| Table 1. SPIRIT chart for stages of assessment in the RCT. | | | | | | | | |
| --- | --- | --- | --- | --- | --- | --- | --- | --- |
|  | Study period | | | | | | | |
|  | Enrolment | Allocation | Post-allocation | | | | | |
| Timepoint | T-1 | T0 | T1 | T2 | T3 | T4 | T5 | T6 |
| Enrolment | | | | | | | | |
| Eligibility screen | X |  |  |  |  |  |  |  |
| Informed consent | X |  |  |  |  |  |  |  |
| Allocation | | | | | | | | |
| Randomization |  | X |  |  |  |  |  |  |
| Treatment group allocation |  | X |  |  |  |  |  |  |
| Intervention | | | | | | | | |
| Group-delivered CBT-I |  |  | X | X |  |  |  |  |
| Waiting list |  |  | X | X | X | X |  |  |
| Assessments | | | | | | | | |
| Baseline questionnaire |  |  | X |  |  |  |  |  |
| Self-reported questionnaire, incl. primary and secondary outcomes |  |  | X | X | X | X |  |  |
| 7-day sleep diary |  |  | X | X | X | X |  |  |
| Process evaluation – quantitative |  |  | X |  | X |  |  |  |
| Process evaluation – qualitative |  |  |  | X | X |  |  |  |
| National registers |  |  |  |  |  |  | X | X |
