# Supplementary material for: Group-delivered cognitive behavioural therapy versus waiting list in the treatment of insomnia in primary care: study protocol for a pragmatic, multicentre randomized controlled trial
Source: BMC Prim Care. 2023 Mar 2;24:61. doi: 10.1186/s12875-023-02018-4 (PMC9979487; doi:10.1186/s12875-023-02018-4)
Supplement: Supplementary file 2 — Additional file 2. [file 12875_2023_2018_MOESM2_ESM.docx]

| S.Table 2. SPIRIT checklist detailing the content of the protocol. | | | |
| --- | --- | --- | --- |
| Section / item | Item |  | Page |
| Administrative information | | | |
| Title | 1 |  | 1 |
| Trial registration | 2a | Trial identifier and registry name. | 2 |
|  | 2b | All items from the World Health Organization Trial Registration Data Set | SF3 |
| Protocol version | 3 |  | 1 |
| Funding | 4 |  | 19 |
| Roles and responsibilities | 5a | Names, affiliations, and roles of protocol contributors | 1, 18 |
|  | 5b | Name and contact information for the trial sponsor | 19 |
|  | 5c | Role of study sponsor and funders, if any, in study design | 19 |
|  | 5d | Composition, roles, and responsibilities of the coordinating centre, steering committee, endpoint adjudication committee, data management team, and other  individuals or groups overseeing the trial, if applicable | 14 |
| Introduction | | | |
| Background and rationale | 6a | Description of research question and justification for undertaking the trial, including summary of relevant studies (published and unpublished) examining  benefits and harms for each intervention | 1 |
|  | 6b | Explanation for choice of comparators |  |
| Objectives | 7 |  | 1-2 |
| Trial design | 8 |  | 1, 5 |
| Methods: Participants, interventions, and outcomes | | | |
| Study setting | 9 |  | 5 |
| Eligibility criteria | 10 |  | 7 |
| Interventions | 11a | Interventions for each group | 8-9 |
|  | 11b | Criteria for discontinuing or modifying allocated interventions for a given trial participant | 7 |
|  | 11c | Strategies to improve adherence to intervention protocols, and any procedures for monitoring adherence | 7 |
|  | 11d | Relevant concomitant care and interventions that are permitted or prohibited during the trial | 9 |
| Outcomes | 12 |  | 9-13 |
| Participant timeline | 13 |  | 6 |
| Sample size | 14 |  | 13 |
| Recruitment | 15 |  | 6-7 |
| Methods: Assignment of interventions (for controlled trials) | | | |
| Sequence generation | 16a | Method of generating the allocation sequence, and list of any factors for stratification. | 7-8 |
| Allocation concealment  mechanism | 16b | Mechanism of implementing the allocation sequence | 7-8 |
| Implementation | 16c | Who will generate the allocation sequence, who will enrol participants, and who will assign participants to interventions | 8 |
| Blinding (masking) | 17a | Who will be blinded after assignment to interventions and how | 7-8 |
|  | 17b | If blinded, circumstances under which unblinding is permissible and procedure for revealing a participant’s allocated intervention during the trial | NA |
| Methods: Data collection, management, and analysis | | | |
| Data collection methods | 18a | Plans for assessment and collection of outcome, baseline, and other trial data, including any related processes to promote data quality along with their reliability and validity, if  known. | 9-10 |
|  | 18b | Plans to promote participant retention and complete follow-up, including list of any outcome data to be collected for participants who discontinue or deviate  from intervention protocols | 7 |
| Data management | 19 |  | 14 |
| Statistical methods | 20a | Statistical methods for analysing primary and secondary outcomes. Reference to where other details of the statistical analysis plan can be found, if not in the  protocol | 14-15 |
|  | 20b | Methods for any additional analyses | 15 |
|  | 20c | Definition of analysis population relating to protocol non-adherence and any statistical methods to handle missing data | 14-15 |
| Methods: Monitoring | | | |
| Data monitoring | 21a | Composition of data monitoring committee (DMC) | 15-16 |
|  | 21b | Description of any interim analyses and stopping guidelines | 15 |
| Harms | 22 |  | 15-16 |
| Auditing | 23 |  | 14 |
| Ethics and dissemination | | | |
| Research ethics approval | 24 |  | 16 |
| Protocol amendments | 25 |  | NA |
| Consent or assent | 26a | Who will obtain informed consent or assent from potential trial participants or authorised surrogates, and how | 7 |
|  | 26b | Additional consent provisions for collection and use of participant data and biological specimens in ancillary studies, if applicable | NA |
| Confidentiality | 27 |  | 14 |
| Declaration of interests | 28 |  | 19 |
| Access to data | 29 |  | 14 |
| Ancillary and post-trial care | 30 |  | 15 |
| Dissemination policy | 31a | Plans for investigators and sponsor to communicate trial results to participants, healthcare professionals, the public, and other relevant groups including any publication restrictions | 16 |
|  | 31b | Authorship eligibility guidelines and any intended use of professional writers | 18 |
|  | 31c | Plans, if any, for granting public access to the full protocol, participant-level dataset, and statistical code | 14 |
| Appendices | | | |
| Informed consent materials | 32 |  | NA |
| Biological specimens | 33 |  | NA |
