# Supplementary material for: Group-delivered cognitive behavioural therapy versus waiting list in the treatment of insomnia in primary care: study protocol for a pragmatic, multicentre randomized controlled trial
Source: BMC Prim Care. 2023 Mar 2;24:61. doi: 10.1186/s12875-023-02018-4 (PMC9979487; doi:10.1186/s12875-023-02018-4)
Supplement: Supplementary file 3 — Additional file 3. [file 12875_2023_2018_MOESM3_ESM.docx]

| S.Table 3. The World Health Organization trial registration data set for this RCT. | |
| --- | --- |
| **Data category** | **Information** |
| Primary registry and trial identifying number | ISRCTN registry, ref. ISRCTN16185698 |
| Date of registration in primary registry | 17/11/2022 |
| Secondary identifying numbers | - |
| Source(s) of monetary or material support | Stiftelsen Dam  Fysiofondet  Trøndelag Fylkeskommune |
| Primary sponsor | Stiftelsen Dam |
| Secondary sponsor(s) | The Norwegian Fund for Postgraduate Training in Physiotherapy  Trøndelag county authority |
| Contact for public queries | MH, ESS |
| Contact for scientific queries | MH, ESS, IM |
| Public title | Effectiveness of group-delivered therapy for insomnia |
| Scientific title | Group-delivered cognitive behavioural therapy for insomnia in primary care |
| Countries of recruitment | Norway |
| Health condition(s) or problem(s) studied | Insomnia |
| Intervention(s) | Group-delivered cognitive behavioral therapy for insomnia vs. waiting list |
| Key inclusion and exclusion criteria | Inclusion: Older than 18 years, Insomnia Severity Index ≥ 12  Exclusion: Not diagnosed with any psychotic or bipolar disorders, personality disorders, dementia or other neurodegenerative diseases. Not affected by heart surgery <3 months, cancer treatment at inclusion, untreated sleep apnea, multiple sclerosis with attack at inclusion |
| Study type | Pragmatic, multicenter randomized controlled trial |
| Date of first enrolment | August 2022 |
| Target sample size | 292 |
| Recruitment status | Recruiting |
| Primary outcome | Insomnia severity measured by Insomnia Severity Index at 3-month follow-up |
| Key secondary outcomes | Health-related quality of life measured by EuroQol EQ5D-5L at 3-month follow-up  Fatigue measured by Chalder Fatigue Scale at 3-month follow-up  Mental distress measured by Hopkins Symptoms Checklist at 3-month follow-up |
